# Observation of positive–negative sub-wavelength interference without intensity correlation calculation

**DOI:** 10.1038/s41598-021-82030-9

**Published:** 2021-01-28

**Authors:** Ling-Yu Dou, De-Zhong Cao, De-Qin Xu, An-Ning Zhang, Xin-Bing Song

**Affiliations:** 1 School of Physics, Beijing Institute of Technology, Beijing, 100081 China; 2 Department of Physics, Yantai University, Yantai, 264005 China; 3 School of Science, Tianjin University of Technology and Education, Tianjin, 300222 China

**Keywords:** Optical physics, Other photonics

## Abstract

We report an experimental demonstration of positive–negative sub-wavelength interference without correlation. Typically, people can achieve sub-wavelength effects with correlation measurement no matter by using bi-photon or thermal light sources. In this paper, we adopt a thermal light source, and we count the realizations in which the intensities of the definite symmetric points are above or below a certain threshold. The distribution of numbers of these realizations which meet the restriction will show a sub-wavelength effect. With proper constrictions, positive and negative interference patterns are demonstrated.

## Introduction

Since the first demonstrations of correlated imaging and interference with entangled sources^1,2^, correlation has been a hot topic in the area of quantum imaging. To achieve a high qualified image, a lot of schemes were explored^3–19^. Besides imaging, interference was widely discussed with correlation method^20–25^. Among all the correlation effects, the sub-wavelength effect has attracted more attention because this effect can break the limit of diffraction^26–28^, which means the resolution can be improved by a factor of 2 in principle. The first sub-wavelength effect was achieved with two-photon quantum source^26^. It is a quantum mechanical two-photon phenomenon but not a violation of the uncertainty principle. Because of the potential application in quantum lithography, this work aroused a hot discussion and hundreds of follow-up studies. The sub-wavelength effect is not unique for quantum sources, and it was soon demonstrated with classical sources^27,28^. In these works, intensity-intensity correlation of the scattered light from a double-slit object was employed at a pair of symmetric positions. Either with a quantum source or with a classical one, the sub-wavelength effect is achieved in second-order correlation in mathematics and shows second-order coherence.

In recent years, positive–negative images were reconstructed through selective averaging of the intensity of a reference detector that had never interacted with the target field^29–31^. Inspired by these works, Wu’s group demonstrated an experiment about thermal light sub-wavelength diffraction using positive and negative correlations^32^. They separated the intensities into two groups by comparing them with their average, and observed a subwavelength diffraction by performing the second-order intensity correlation in opposite directions.

In the previous works, the sub-wavelength effect was achieved by coincidence measurement with two-photon source or correlation measurement with a thermal field, i.e., second-order correlation. However, it is not necessary to achieve a sub-wavelength effect by second-order correlation. In this paper, we demonstrate a scheme to show sub-wavelength interference without correlation. We set thresholds for the detector, and count the realizations when the signals are above or below the thresholds. Positive sub-wavelength interference appears when the intensities are synchronously above or below their thresholds at a pair of symmetric positions. When one intensity is above its threshold and the other intensity is below the threshold at the symmetric position, a negative sub-wavelength interference appears. We also demonstrate the relation between the visibility and thresholds.

## Results and discussion

**Figure 1:**
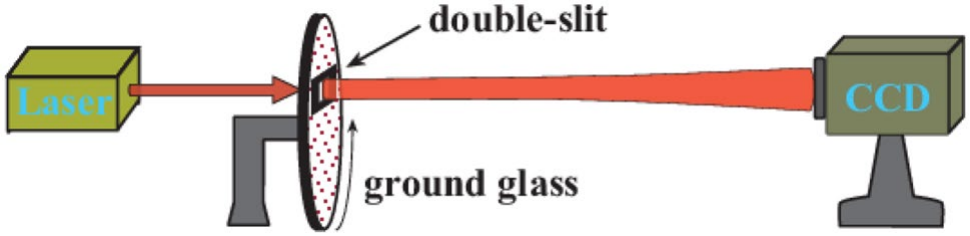
Experimental setup. A laser beam passes through a rotating ground glass to generate pseudo-thermal light. A double-slit is placed after the ground glass. A CCD camera records the speckles scattered from the double-slit.

The setup is depicted in Fig. 1. A He–Ne laser beam with wavelength λ = 632.8 nm impinges on a slowly rotating ground glass with a rotation frequency of $2 \times 10^{ - 3}$ Hz to form a pseudo-thermal light source. A double-slit (150 μm width and 300 μm separation, center to center) is placed directly after the ground glass. The light is recorded by a charge-coupled device (CCD, whose output intensity varies from 0 to 255) camera. In our experiment, the laser beam has a diameter of 3 mm on the ground glass. The distance from the double-slit to the CCD is 40 cm. A total of 10,000 realizations (measurements) were adopted.

If we mark the intensity of position *x* on the CCD plane as $I(x)$, and we calculate the intensity correlation between a fixed position $x_{0}$ and the whole plane, then a classical diffraction pattern can be observed, like

1 $$g_{cc}^{(2)} (x) = \frac{{\left\langle {I(x_{0} )I(x)} \right\rangle }}{{\left\langle {I(x_{0} )} \right\rangle \left\langle {I(x)} \right\rangle }} = 1 + \frac{{\left\langle {\Delta I(x_{0} )\Delta I(x)} \right\rangle }}{{\left\langle {I(x_{0} )} \right\rangle \left\langle {I(x)} \right\rangle }},$$

where $\left\langle \cdot \right\rangle$ means ensemble average, and $\Delta I = I - \left\langle I \right\rangle$.

When the correlation is between symmetric positions (*x* and − *x*), a sub-wavelength effect appears

2 $$g_{sub}^{(2)} (x) = \frac{{\left\langle {I(x)I( - x)} \right\rangle }}{{\left\langle {I(x)} \right\rangle \left\langle {I( - x)} \right\rangle }} = 1 + \frac{{\left\langle {\Delta I(x)\Delta I( - x)} \right\rangle }}{{\left\langle {I(x)} \right\rangle \left\langle {I( - x)} \right\rangle }}.$$

Because of the background in the above two correlations, the contrast has a maximum of 1/3 in principle^28^.

**Figure 2:**
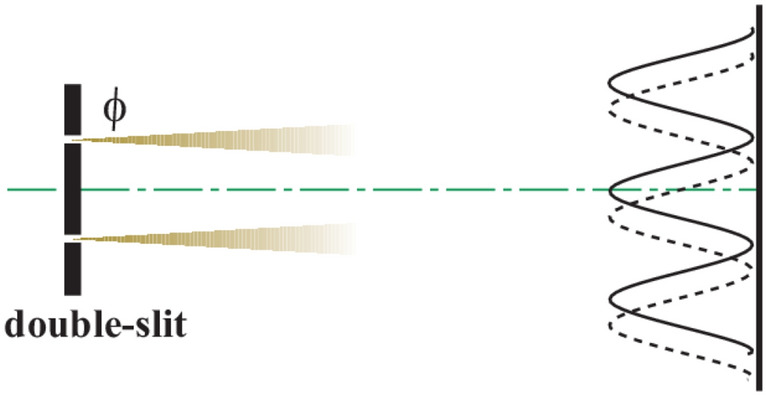
Sketch of the random phase interference model. The intensities from the two slits are assumed equal, and the phase of light from the upper slit is random.

It seems widely known that the sub-wavelength effect can be observed in a second-order correlation. However, we can show that, without correlation, a sub-wavelength effect can be achieved. To avoid the complexity in statistical theory, here we choose an ideal and simple model to explain this effect qualitatively. We assume that the light passes through the double slits with equal intensities and generates random centered interference fringes, as shown in Fig. 2. The model can be like this, for each realization there being a random phase $\phi$ in the upper slit and the diffraction fringes shift randomly. Here we assume that the phase $\phi$ has a uniform distribution between 0 and $2\pi$. Apparently, we can write the fringe distribution as $I(\tilde{x},\phi ) = \frac{{\cos (\tilde{x} + \phi ) + 1}}{2} = \cos^{2} \left( {\frac{{\tilde{x} + \phi }}{2}} \right)$. Here $\tilde{x} = 2\pi \frac{x}{\Lambda }$ is the normalized position coordinate and $\Lambda$ is the period of classical diffraction fringes. For simplicity, we normalize the maximum value of $I$ to 1. When we consider the intensities of symmetric positions ($\tilde{x}$ and $- \tilde{x}$) above some threshold value $I_{{{\text{th}}}}$, we have

3 $$I(\tilde{x},\phi ) = \cos^{2} \left( {\frac{{\tilde{x} + \phi }}{2}} \right) \ge I_{{{\text{th}}}} ,$$

4 $$I( - \tilde{x},\phi ) = \cos^{2} \left( {\frac{{ - \tilde{x} + \phi }}{2}} \right) \ge I_{{{\text{th}}}} ,$$

This means that the probability of both the two intensities above $I_{{{\text{th}}}}$ is the weight of $\phi$ which make Eqs. (3) and (4) satisfied simultaneously. We know that the period of the fringe of intensity is $2\pi$. Now let us consider the period of the distribution for the situation of the intensities at symmetric positions above the threshold simultaneously. From the experimental results, we can find that the periods of them are half of the period of intensity diffraction pattern. Here we give a simple explanation. When we set $\tilde{x} = \tilde{x^{\prime}} + \pi$, we will have $I(\tilde{x},\phi ) = \cos^{2} \left( {\frac{{\tilde{x}^{\prime} + \phi + \pi }}{2}} \right)$ and $I( - \tilde{x},\phi ) = \cos^{2} \left( {\frac{{ - \tilde{x}^{\prime} + \phi - \pi }}{2}} \right)$. If we set $\phi^{\prime} = \phi + \pi$, we have $I(\tilde{x},\phi ) = \cos^{2} \left( {\frac{{\tilde{x}^{\prime} + \phi^{\prime}}}{2}} \right) = I(\tilde{x}^{\prime},\phi^{\prime})$ and $I( - \tilde{x},\phi ) = \cos^{2} \left( {\frac{{ - \tilde{x}^{\prime} + \phi^{\prime} - 2\pi }}{2}} \right) = I( - \tilde{x}^{\prime},\phi^{\prime})$. So $\pi$ is the period of the distribution for the situation of the intensities at symmetric positions above the threshold simultaneously. Apparently, it is half of the period for classical diffraction fringes.

**Figure 3:**
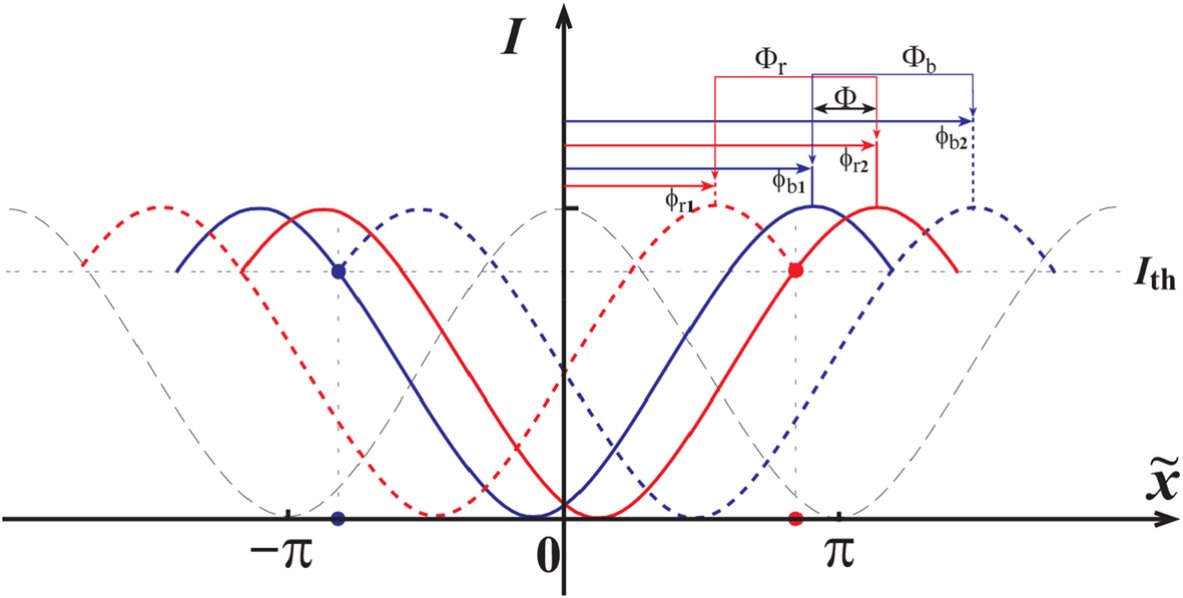
Interference curves for different phase differences. Black dash line is the interference curve for $\phi = 0$. Red dash and real lines demonstrate the range of intensity of red spot above the threshold intensity. Blue dashed and real lines demonstrate the range of intensity of blue spot above the threshold intensity.

**Figure 4:**
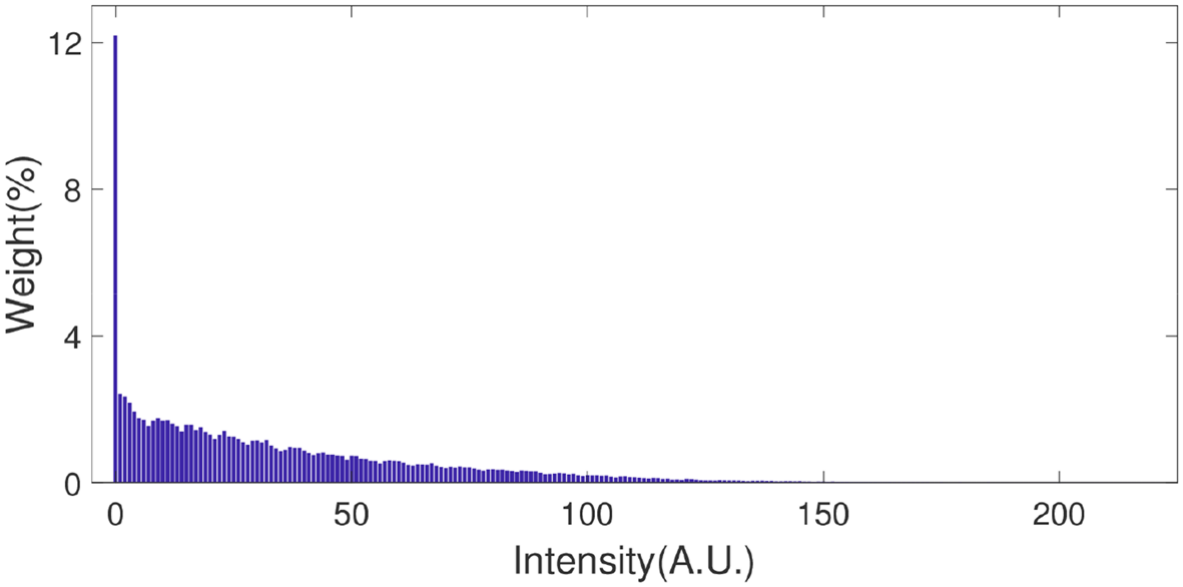
Intensity statistical distribution.

In Fig. 3, we give a detailed analysis when the intensities at two symmetric positions are above the threshold intensity $I_{{{\text{th}}}}$. For other cases, the analysis is similar. As shown in Fig. 3, the symmetric positions $\tilde{x^{\prime}}$ and $- \tilde{x^{\prime}}$ are marked as red spots and blue spots, respectively. The threshold intensity $I_{{{\text{th}}}}$ is marked with a horizontal dashed line. The black dashed line demonstrates the diffraction curve when $\phi = 0$. When the phase shifts from $\phi = \phi_{{{\text{r1}}}}$ to $\phi = \phi_{{{\text{r2}}}}$, the intensity at the position of the red spot ($\tilde{x^{\prime}}$) is above $I_{{{\text{th}}}}$. So, $\Phi_{{\text{r}}}$ is the range that make Eq. (3) satisfied. It is not hard to find out that $\phi_{{{\text{r1}}}} = \tilde{x^{\prime}} - \cos^{ - 1} (2I_{{{\text{th}}}} - 1)$ and $\phi_{{{\text{r2}}}} = \tilde{x^{\prime}} + \cos^{ - 1} (2I_{{{\text{th}}}} - 1)$, where $\cos^{ - 1}$ is an inverse trigonometric function. Similarly, we can find the range $\Phi_{{\text{b}}}$ for the blue spot. So, the widths of $\Phi_{{\text{r}}}$ and $\Phi_{{\text{b}}}$ are same, i.e. $2\cos^{ - 1} (2I_{{{\text{th}}}} - 1)$, which we marked as $\Phi_{{{\text{th}}}}$. The overlap between $\Phi_{{\text{r}}}$ and $\Phi_{{\text{b}}}$, marked as $\Phi$ ($0 \le \Phi \le 2\pi$), is the phase range which makes Eqs. (3) and (4) satisfied simultaneously. It is not hard to conclude that when $I_{{{\text{th}}}}$ is increased the range of $\Phi$ is smaller and the visibility increases. For this simple model, we can give an approximate expression about the visibility as 1 for $0 \le \Phi_{{{\text{th}}}} \le \pi$, and $\frac{{2\pi - \Phi_{{{\text{th}}}} }}{{3\Phi_{{{\text{th}}}} - 2\pi }}$ for $\pi \le \Phi_{{{\text{th}}}} \le 2\pi$. However, our experimental source is definitely different from this ideal model. This model will give a homogeneous distribution when we check the intensities at a certain pixel on the camera. But, in our experiment, the intensities have a quasi-negative exponential probability distribution, as shown in Fig. 4. Although the analytical formula is not precise for our experiment, the conclusion about the visibility is agree with our experimental results qualitatively, as shown in Fig. 6a–d.

**Figure 5:**
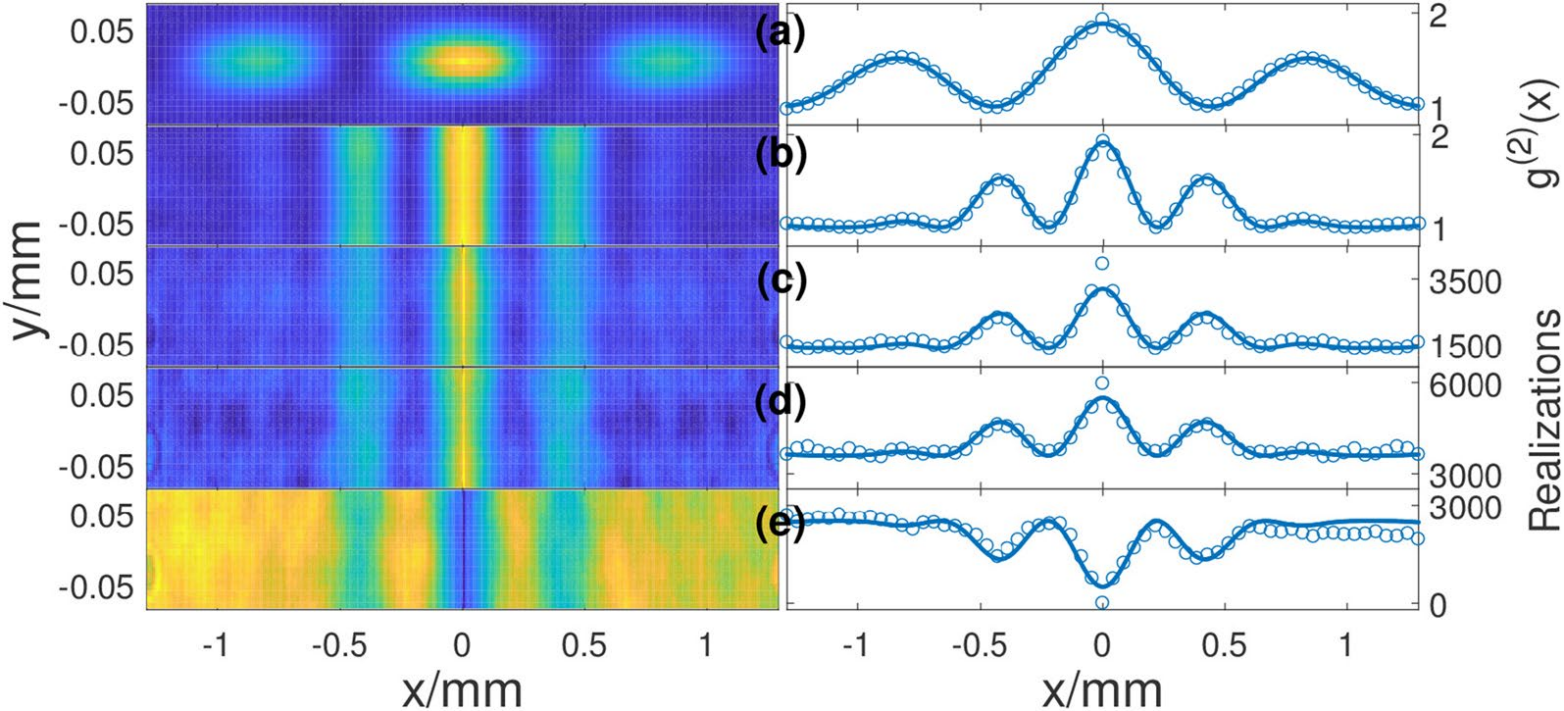
Experimental results. Left column shows the 2-D results and right column shows the corresponding cross section results. In the right column, open circles are experimental results and solid curves are theoretical fitting. (**a**) Normalized second-order correlation between a fixed position (center) and the whole plane. (**b**) Sub-wavelength correlation. The labels of Y-axis for (**a**) and (**b**) are marked as $g^{(2)} (x)$. (**c**) The realizations when intensities at symmetric positions (*x* and − *x*) above the average intensity. Similarly, the realizations for the intensities at *x* and − *x* below the average intensity are shown in (**d**). (**e**) Negative fringe when the intensity at *x* greater than the average intensity and the intensity at − *x* smaller than the average intensity. The labels of Y-axis for (**c**)–(**e**) are marked as Realizations.

Figure 5 depicts our experimental results. The left column shows the 2-D results and the right column shows the corresponding cross-section results. Figure 5a is the normalized second-order correlation between a fixed position (center) and the whole plane. The results, as expected, are like the classical diffraction patterns. Figure 5b is the sub-wavelength correlation, and we can find that the width of the fringe is half of the width of the fringe in Fig. 5a. These results are well known for over ten years. The last three rows are the results when we group the realizations. We counted the realizations when the intensities of symmetric positions (*x* and − *x*) above the average intensity (which is the threshold intensity $I_{{{\text{th}}}}$ for this case) simultaneously, and the results are shown in Fig. 5c. We can find that the distribution of the fringe is nearly the same as Fig. 5b by ignoring the difference of amplitude. Similarly, the distributions of realizations for the intensities at *x* and − *x* below the average intensity are shown in Fig. 5d. A positive fringe pattern appears for this case. This is because the intensities on peak positions are synchronism. Figure 5e shows negative fringes when we set the intensity at *x* greater than the average intensity and the intensity at − *x* smaller than the average intensity.

**Figure 6:**
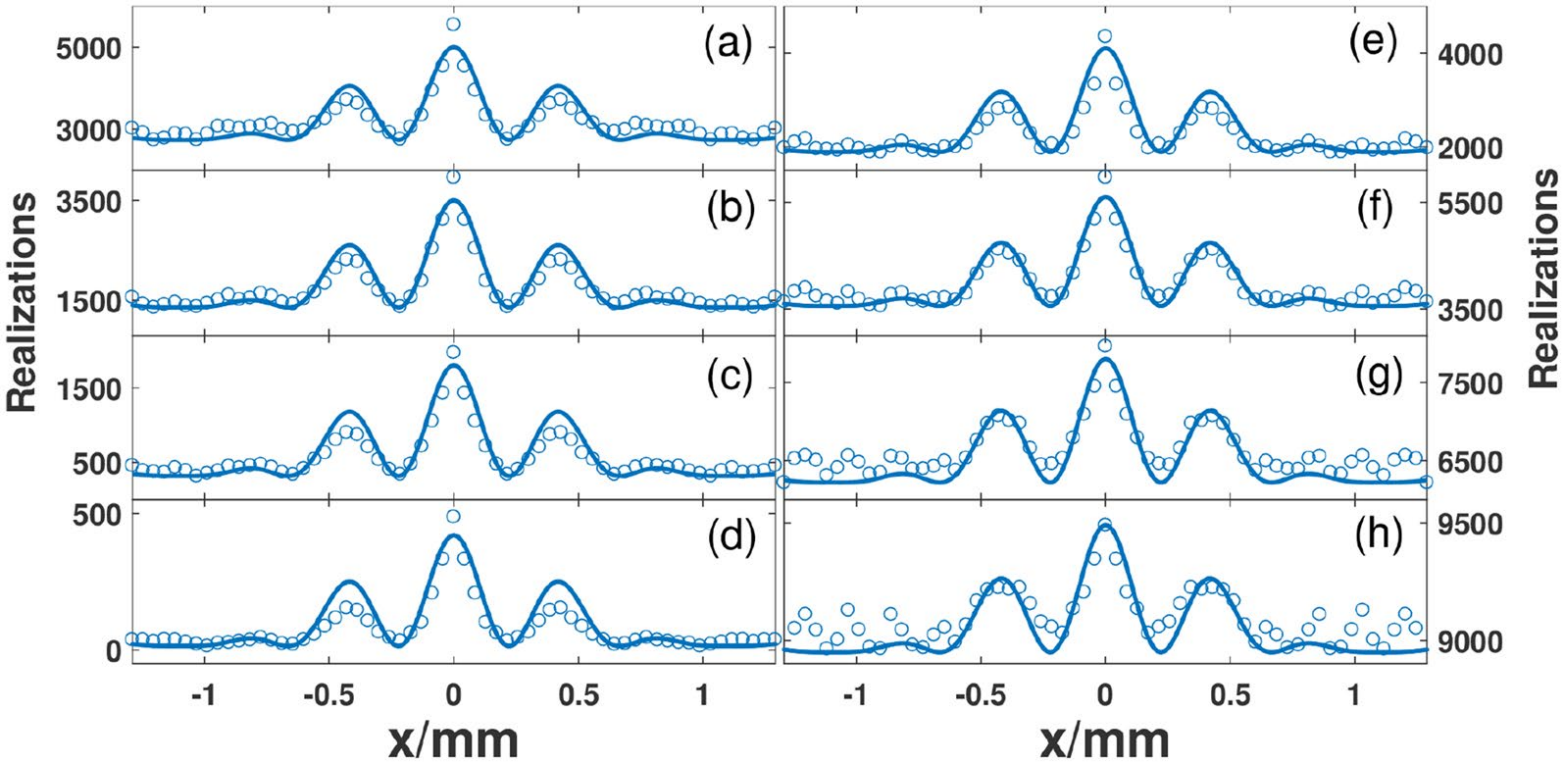
Experimental results with different thresholds. Left column and right column show the results for the cases of intensities at symmetric positions greater and smaller than thresholds, respectively. From top to bottom, the thresholds are set to 20, 34, 60 and 100, respectively. The visibilities are 0.34, 0.49, 0.72 and 0.94 for (**a**)–(**d**), respectively. The visibilities are 0.39, 0.25, 0.12 and 0.033 for (**e**)–(**h**), respectively.

We have demonstrated the sub-wavelength pattern without correlation. Next, we explored the visibility of the fringes qualitatively. In the experiment, we took 10,000 realizations and the average intensity is about 34 (arbitrary unit). The details are shown in Fig. 6. Figure 6a–d are the results for the cases of intensities at symmetric positions greater than 20, 34, 60 and 100, respectively. And the visibilities are 0.34, 0.49, 0.72 and 0.94, respectively. Here, the visibility is defined as ${{(r_{\max } - r_{\min } )} \mathord{\left/ {\vphantom {{(r_{\max } - r_{\min } )} {(r_{\max } + r_{\min } )}}} \right. \kern-\nulldelimiterspace} {(r_{\max } + r_{\min } )}}$, and $r_{\max /\min }$ is the maximum/minimum of the realizations. Similarly, when the intensities at symmetric positions are smaller than 20, 34, 60 and 100, the corresponding curves are shown in Fig. 6e–h, and the corresponding visibilities are 0.39, 0.25, 0.12 and 0.033, respectively. It can be seen that when the threshold is higher the visibility becomes greater for the cases of intensities at symmetric positions greater than the thresholds. Inversely for the cases of intensities at symmetric positions smaller than the thresholds, a higher threshold leads to lower visibility.

## Conclusion

In summary, we have experimentally studied the sub-wavelength effect without correlation. We counted the realizations according the intensities at symmetrical positions above or below some threshold, and we got positive and also negative patterns. Although positive and negative sub-wavelength diffractions were demonstrated recently^32^, the experiment was based on the second-order correlation. Until now, to our knowledge, all the sub-wavelength effects were based on correlation calculation, but our results showed that correlation calculation is not necessary for the sub-wavelength effect. Our sub-wavelength patterns reflect conditional joint statistical probabilities of scattered thermal light field. This experiment was fundamentally important because of the often asked question: What on earth is the coherence of thermal light? We wish our work can deepen the understanding of the sub-wavelength effect. This experiment was practically important because it provides a protocol to get sub-wavelength diffraction information with logic detectors (true/false). This could be extremely useful for long-distance image processing and signal processing with broadband spectrum. This proposal is believed to expand the applicability to enhance precision in measurement and optical lithography.

## Methods

The sub-wavelength effect demonstrated in the work is based on the statistics of the realizations. Although the setup is exactly the same as the setup for the second-order correlation sub-wavelength, the methods is completely different from the second-order (intensity-intensity) correlation. Because of the synchronicity of the intensities at certain symmetric positions, we can sort the realizations according to the intensities at the symmetrical positions above or below some threshold, and get positive and also negative sub-wavelength patterns. In our experiment, we recorded a series of speckles patterns, then we compared the intensities at symmetrical pixels with a threshold ($I_{{{\text{th}}}}$) for every pattern. If the intensities satisfy the corresponding restriction for a pattern, we count a valid realization at symmetrical pixels.
